# Biocomposite Microparticles Incorporating Ibuprofen and ZnO Nanoparticles for Controlled Drug Delivery and Antimicrobial Therapy

**DOI:** 10.3390/polym18192329

**Published:** 2026-09-24

**Authors:** Delia Mihaela Rata, Gisela Trifas, Raluca Somoghi, Anca Niculina Cadinoiu, Doina Simona Matiut, Cosmin Teodor Mihai, Oana-Maria Daraba, Ketan Kuperkar, Leonard Ionut Atanase

**Affiliations:** 1 Faculty of Medicine, “Apollonia” University of Iasi, 700511 Iasi, Romania; delia.rata@univapollonia.ro (D.M.R.); oana.daraba@univapollonia.ro (O.-M.D.); 2 “Cristofor Simionescu” Faculty of Chemical Engineering and Environmental Protection, “Gheorghe Asachi” Technical University of Iasi, 700050 Iasi, Romania; gisela_trifas@yahoo.com; 3 Faculty of Petroleum Refining and Petrochemistry, Petroleum-Gas University of Ploiesti, 100680 Ploiesti, Romania; r.somoghi@gmail.com; 4 National Institute for Research and Development in Chemistry and Petrochemistry-ICECHIM, 060021 Bucharest, Romania; 5 Medical Investigations Praxis SRL, 700376 Iasi, Romania; smatiut@yahoo.com (D.S.M.); cosmin-teodor.mihai@laboratorpraxis.ro (C.T.M.); 6 Faculty of Biology, “Alexandru Ioan Cuza” University of Iasi, 700506 Iasi, Romania; 7 Department of Chemistry, Sardar Vallabhbhai National Institute of Technology (SVNIT), Ichchhanath, Surat 395007, Gujarat, India; kck@chem.svnit.ac.in; 8 Academy of Romanian Scientists, 010071 Bucharest, Romania

**Keywords:** polymeric microparticles, drug delivery, hemocompatibility, cell viability, antimicrobial activity

## Abstract

Preparation of complex biomaterials capable of providing both controlled drug delivery and an increased antimicrobial effect without affecting healthy cells remains a major challenge in pharmaceutical and biomedical applications. This study reports the successful preparation of biocomposite microparticles based on carboxymethyl chitosan and poly (vinyl alcohol), co-loaded with zinc oxide nanoparticles with antimicrobial effect and Ibuprofen with anti-inflammatory, antipyretic, and analgesic effect. An environmentally friendly water-in-oil emulsion method using DL-limonene as a biocompatible oil phase was used to obtain these microparticles. FTIR analysis confirmed the formation of intermolecular ester and amide bonds and the successful incorporation of zinc oxide nanoparticles into the polymeric matrix. The obtained microparticles exhibited diameters ranging from 533 to 1054 nm and provided a biphasic Ibuprofen release profile, characterized by an initial burst release within the first 3 h followed by sustained diffusion-controlled release, achieving cumulative drug release of 84–100%. Biocomposite microparticles presented excellent hemocompatibility, with hemolysis values between 0.41% and 2.47%. Moreover, after 24 h of exposure to microparticles, human dermal fibroblasts exhibited a cellular viability ranging from 82.24% to 94.22% whereas prolonged treatment for 48 h resulted in a slight decrease of cell viability until 74.88%. Biocomposite microparticles exhibited notable antimicrobial activity against *Streptococcus pneumoniae* strain. These findings demonstrate that the combination of green fabrication, controlled Ibuprofen release, good biocompatibility, and antimicrobial efficacy highlights the potential of these biocomposite microparticles as innovative hybrid nanostructures that can significantly improve therapeutic outcomes.

## 1. Introduction

Development of complex microparticles that simultaneously prevent bacterial colonization, control inflammation, and ensure a good biocompatibility has recently been explored in modern medicine and especially in drug delivery applications [1]. Conventional therapy is generally based on the administration of antimicrobial and anti-inflammatory agents in the form of separate formulations, which can lead to inconveniences in terms of dosage frequency, immediate release of the active principle, low patient compliance, and, sometimes, high cost. In this context, biomaterial-based delivery platforms that integrate different therapeutic agents into a single system have emerged as promising alternatives for improving treatment efficacy while minimizing systemic side effects [2]. Polymeric microparticles are particularly attractive for localized drug delivery due to enhanced pharmacokinetics and stability of the therapeutic agents against possible enzymatic and/or hydrolytic degradation. Moreover, they can successfully incorporate both hydrophobic and hydrophilic drugs, pharmaceutical proteins, and also inorganic nanomaterials (such as gold, silver, zinc oxide, and magnetic nanoparticles) to achieve a high therapeutic effect [3]. Among natural polymers, chitosan has garnered considerable attention owing to its biodegradability, biocompatibility, low toxicity, and intrinsic biological activity. Chitosan is the second-most prevalent biopolymer in nature after cellulose and has a wide application due to the amine and hydroxyl functional groups in its structure. However, its solubility restricted to acidic media represents a significant limitation to its broader application [4]. Significant advances have been made in the chemical modification of chitosan, leading to the synthesis of derivatives with improved water solubility. Among these derivatives, carboxymethyl chitosan (CMCS) is the most studied because it can be easily synthesized and has a high application potential [5]. CMCS is obtained by substituting hydroxyl groups in the structural units of glucosamine with carboxymethyl groups and has recently attracted great interest due to its increased water solubility and high biocompatibility [6]. Due to these characteristics, CMCS has shown interest in drug delivery in various forms, such as films, hydrogels, fibers, or particulate systems [7].

Antimicrobial resistance represents a critical global health challenge, and it is estimated that by 2050, there will be over 39 million deaths caused by antibiotic-resistant infections [8]. The challenge of this acquired resistance of microorganisms to classical antimicrobial drugs has led in the last decades to the exploration of new strategies to improve antimicrobial treatments [9,10,11,12]. The use of non-antibiotic antimicrobial agents, such as silver, zinc oxide (ZnO), or titanium dioxide (TiO_2_) nanoparticles exert bactericidal effects through disruption of the cell membrane, thereby circumventing the emergence of drug resistance [13,14,15]. Embedding ZnO nanoparticles (ZnONPs) into polymeric microparticles can provide improved mechanical strength, biocompatibility, and enhanced functionality [16,17]. ZnONPs have been studied for the development of the next generation of nanoantibiotics against pathogenic microorganisms to combat multidrug resistance. They are of particular interest due to several unique properties, including non-toxicity, availability, stability, strong antibacterial, and anti-inflammatory activities [18,19,20,21,22,23]. Complementarily, an anti-inflammatory agent targeting symptomatic manifestations of infection may be co-incorporated alongside the antimicrobial component into delivery systems, which can control symptoms such as pain, inflammation, or fever. Ibuprofen is one of the most well-known nonsteroidal anti-inflammatory drugs (NSAIDs), being widely used in therapy due to its anti-inflammatory, analgesic, and antipyretic effects [24,25].

In this study, we hypothesized that carboxymethyl chitosan/polyvinyl alcohol-based biocomposite microparticles, synthesized through a green water/oil emulsion method and co-loaded with Ibuprofen and ZnONPs, would generate stable, biocompatible particulate systems capable of sustained drug release for the treatment of bacterial infections.

Polyvinyl alcohol (PVA) is a biodegradable, biocompatible, and non-toxic semi-crystalline synthetic polymer that has been used for many years in the manufacture of various drug delivery systems such as hydrogels, microparticles and nanoparticles [26,27]. Mixing PVA with CMCS increases the stability, biodegradability, and biocompatibility of the obtaining systems. Various CMCS/PVA delivery systems have previously been reported, but most of these rely on conventional crosslinking agents such as glutaraldehyde, epichlorhydrin, or carbodiimide-based chemicals, or they require organic solvents during fabrication [28,29,30,31]. Although these strategies provide satisfactory mechanical stability, they may leave residual toxic reagents, require extensive purification, or involve stringent processing conditions that limit their biomedical applicability. Therefore, there is still a need for CMCS/PVA-based microparticle systems prepared using milder and more environmentally benign synthesis routes while maintaining adequate structural integrity and drug delivery performance.

In this study, DL-limonene, a renewable terpene extracted from citrus peels, was selected as the oil phase for the water/oil emulsion. In addition to replacing petroleum-derived organic solvents, DL-limonene has intrinsic antimicrobial and anticancer activities and is already used in pharmaceutical, cosmetic, and food applications [32]. In addition, the coupling reagent 4-(4,6-dimethoxy-1,3,5-triazin-2-yl)-4-methylmorpholinium chloride (DMT-MM) was used to promote ester bond formation between CMCS and PVA under mild aqueous conditions [33]. Unlike conventional covalent crosslinking systems, this approach avoids the use of highly toxic crosslinking agents while producing stable polymer networks with improved resistance to premature degradation.

Therefore, the novelty of the present study does not lie exclusively in the use of an environmentally friendly synthesis method, but in the combination of several complementary features that, to the best of our knowledge, have not been reported together for CMCS/PVA microparticles: (i) a water/oil emulsion process using bio-based DL-limonene as the oil phase, (ii) facile DMT-MM-mediated esterification without conventional toxic crosslinking agents, and (iii) the development of a multifunctional CMCS/PVA carrier that simultaneously incorporates ibuprofen and ZnO nanoparticles to ensure the controlled release of anti-inflammatory drugs, along with an intrinsic antibacterial activity. This integrated strategy provides a sustainable alternative to previously reported CMCS/PVA microcarrier systems while extending their therapeutic functionality.

The obtained microparticles were structurally characterized by FTIR analysis. The average diameter and particle size distribution of the microparticles were determined by dynamic light scattering (DLS) analyses, whereas their morphology was evaluated by transmission electron microscopy (TEM). SEM-EDAX analysis was used to determine the amount of ZnONPs incorporated into the biocomposite microparticles. The stability of the biocomposite microparticles in aqueous solution simulating the biological environment (PBS; pH 7.4) was investigated by electrophoresis at 25 °C using the Zeta Nanosizer Malvern. The swelling degree, loading efficiency, and the release kinetics of Ibuprofen from the microparticles in solution-simulating biological fluids (PBS with pH 7.4) were also studied. Stability tests of the microparticles in PBS solution and hemolysis test were performed to evaluate the hemolytic potential of the obtained microparticles. Moreover, the cellular viability on human dermal fibroblast and antimicrobial activity against *Streptococcus pneumoniae* strain were carried out.

The major significance of this study is the development of a complex therapeutic pharmaceutical product that combines an anti-inflammatory drug with an inorganic antimicrobial agent using a sustainable preparation strategy. Ibuprofen was selected as a model anti-inflammatory drug because, in addition to its established anti-inflammatory activity, its potential for pulmonary administration has been investigated in recent studies. These studies support the feasibility of using ibuprofen in particulate systems for pulmonary and intranasal drug delivery, demonstrating efficient encapsulation, sustained release, and suitable aerodynamic and dissolution properties [34,35,36]. The long-term objective of this research is to develop a biocompatible, biodegradable, and non-toxic polymeric microparticulate system with potential for local delivery of active pharmaceutical ingredients to the respiratory tract either by aerosolization or intranasal administration for the future management of pulmonary infections. Further studies will evaluate the specific characteristics required for pulmonary delivery, including biological safety, aerosolization performance, and therapeutic efficacy.

## 2. Materials and Methods

### 2.1. Materials

CMCS (Mw = 75 KDa and 95% substitution degree of O-carboxymethylation) with almost unsubstituted amino group and deacetylation degree ≥ 90% was purchased from Santa Cruz Biotechnology (Dallas, TX, USA). PVA (Mw = 9000–10,000 g/mol, 80% hydrolyzed); Ibuprofen sodium salt, acetone, Tween 80, Span 80, and Triton X 100 were purchased from Sigma-Aldrich (St. Louis, MO, USA). ZnONPs with diameter of 10–30 nm were purchased from US Research Nanomaterials, Houston, TX, USA. D-(+)-Limonen and PreMix PBS were acquired from Carl Roth, whereas the coupling agent DMT-MM was procured from Alfa Aesar. All reagents used for the cytotoxicity test like Human dermal fibroblasts (HDFa), fetal bovine serum (FBS), non-essential amino acids, Dulbecco’s Modified Eagle Medium (DMEM), antibiotics (streptomycin/penicillin), and trypsin-EDTA were purchased from Thermo Fisher Scientific (Waltham, MA, USA). Ultrapure water necessary for synthesis and analysis was produced in our laboratory.

### 2.2. Microparticles Preparation

The microparticles were prepared by a simple water/oil (W/O) emulsion process, according to Figure 1, involving two different chemical reactions: (i) intermolecular esterification between the carboxyl groups of CMCS and the hydroxyl groups of PVA, as well as (ii) intramolecular amidation between the amine groups with the carboxyl groups of CMCS chains. For both reactions, the DMT-MM activator was used as a coupling agent. Specific amounts of CMCS and PVA were solubilized in 40 mL distilled water at 50 °C (Table 1). Upon complete dissolution of the polymers, 2% (*w*/*w*) Tween 80 was added and stirring was continued for 2 h. Different amounts of ZnONPs were dispersed in 2 mL ultrapure water containing 2% (*w*/*w*) Tween 80, which was sonicated for 2 min. The obtained suspension was introduced into the aqueous polymer solution under continuous magnetic stirring and was kept under agitation for 2 min. Separately, a volume of 5 mL of DMT-MM activator water solution was prepared. The oily phase was formed from Limonene with 2% Span 80 (*w*/*w*). The emulsion was prepared by adding the aqueous phase dropwise into the oily phase under homogenization with an Ultraturrax (T-18) (IKA, Staufen, Germany) at a speed of 9000 rpm. Once the emulsion was formed, homogenization was carried out for additional 15 min at 12,000 rpm. Then, the activator solution was added dropwise and the homogenization continued for 10 more minutes. After this period, the autoclavable screw-top bottle containing the emulsion was moved to a magnetic stirring plate, covered with a tightly sealed and kept for 24 h at room temperature under agitation. The emulsification conditions used in the present study were based on a previously optimized methodology developed by our research team. After 24 h, the emulsion was centrifuged, the supernatant was removed, and the recovered microparticles were extensively alternative washed with acetone and water to remove the oil phase, thus minimizing any residual DL-limonene. The final washing step was performed with acetone; therefore, the recovered microparticles were dried directly from the acetone suspension at room temperature until constant weight.

### 2.3. Characterization Methods

#### 2.3.1. Structural Characterization

The chemical structure of the obtained MPs and MPsZO microparticles was analyzed by FT-IR spectroscopy using a Shimadzu IRSpirit spectrometer (Shimadzu, Kyoto, Japan) equipped with a single-reflection ATR accessory, QATR-S. The spectra were recorded at room temperature, in absorbance mode, in the range of 400–4000 cm^−1^, at a resolution of 4 cm^−1^.

#### 2.3.2. Microparticles Mean Diameter and Size Distribution

The average diameters and the size distribution of the obtained microparticles, after purification and drying, were determined using the dynamic light scattering (DLS) technique. For these analyses, carried out using a Zetasizer Nano ZS from Malvern Panalytical (Malvern, UK), microparticles suspensions were prepared, at a concentration of 0.1% (*w*/*v*) and 25 °C, both in acetone, in order to evaluate their size in the unswollen state, and in four different aqueous media (ultrapure water, saline solution, PBS (pH = 7.4), and FBS 10%), to evaluate their size as closely as possible to the physiological environment. Five successive measurements were carried out for each sample.

#### 2.3.3. Zeta Potential Measurements

The zeta potential, a key parameter that highlights electrostatic interactions in particles suspensions, was measured by laser Doppler electrophoresis at 25 °C (Zetasizer Nano ZS from Malvern Panalytical). Microparticle suspensions in different aqueous solutions (saline, PBS, and 10% FBS solution) were prepared at a concentration of 0.1% *w*/*v*, and zeta potential determinations were performed in triplicate.

#### 2.3.4. Morphology and Elemental Composition of Microparticles

The morphology of the samples was characterized by transmission electron microscope Tecnai™ G2 F20 TWIN Cryo-TEM, FEI Company™ (Hillsboro, OR, USA) operated under an acceleration voltage of 200 kV. A small drop of well dispersed sample, ultrasonicated for 5 min, was put on a carbon film copper grid and then visualized on TEM.

A high-resolution Scanning Electron Microscope (Verios G4 UC from Thermo Scientific, Waltham, MA, USA) equipped with an EDAX module was used to evaluate the elemental composition of the microparticles and the presence of ZnONPs.

#### 2.3.5. Microparticles Behavior in Aqueous Solutions

Since the obtained microparticulate systems were designed for use in biomedical applications, evaluating their swelling behavior in solutions that mimic biological fluids was considered necessary. For this, PBS solution with pH = 7.4 was used to determine the swelling degree by the gravimetric method. Briefly, 20 mg of microparticles were weighed into Eppendorf tubes, to which 2 mL of PBS solution were added. The obtained suspension was maintained at 37 °C under gentle agitation for 24 h. At predetermined times, the suspension was centrifuged, the supernatant was carefully removed, and the equivalent swelling degree was calculated by the ratio between the mass of swollen microparticles and the initial mass of the particles (in dry state) multiplied by 100.

#### 2.3.6. Microparticles Stability Studies

The stability of the microparticles was determined in an aqueous medium (PBS solution at pH 7.4), which is similar to the physiological environment. For these tests, 20 mg of microparticles were carefully weighed and placed in contact with PBS medium. Each sample was kept under gentle agitation at 37 °C for 1 week. After this period, the samples were centrifuged, washed several times with distilled water, and dried in an oven at 37 °C. The tests were performed in triplicate. The stability of the microparticles was calculated indirectly by determining the mass loss (Δm) after 1 week. The mass loss was calculated using the Equation (1):

(1) $$∆m\%=\frac{m_{1}-m_{2}}{m_{1}}\times 100$$

where m_1_ represents the mass of the sample before starting the test (20 mg) and m_2_ represents the mass of the dried sample after degradation.

#### 2.3.7. Loading of Microparticles with Ibuprofen

To evaluate the loading capacity of the microparticles, 20 mg of each sample were first accurately weighed. Then, a stock solution of Ibuprofen in ultrapure water with a concentration of 100 mg/mL was prepared. Further, 1 mL of drug solution was added to Eppendorf tubes (Eppendorf, Hamburg, Germany) together with the previously weighed microparticle samples and was maintained at 37 °C for 24 h under gentle magnetic stirring. The microparticles loaded with Ibuprofen were separated from the supernatant using a Hettich Universal 320 centrifuge. The amount of drug in the supernatant was measured using a Nanodrop One spectrophotometer from Thermo Scientific (Waltham, MA, USA) at a wavelength of 223 nm. Previously, a calibration curve was prepared using standard Ibuprofen solutions in ultrapure water at six concentrations, ranging from 3.125 to 50 μg/mL. The encapsulation efficiency of Ibuprofen in microparticles was calculated as a percentage of (initial drug amount − drug amount in the supernatant)/initial drug amount.

#### 2.3.8. In Vitro Release Studies of Ibuprofen

To evaluate the in vitro release kinetics of Ibuprofen, a dialysis method coupled with UV-Vis spectrophotometric measurement was used. Briefly, 20 mg of Ibuprofen-loaded microparticles were introduced into dialysis tubes, and 2 mL of PBS (pH 7.4) were added over the dried samples. The dialysis tubes were carefully sealed before being fixed onto the stirring paddles of the USP II system. When the experiment started, the paddles carrying dialysis tubes were immersed in 200 mL of PBS solution at pH 7.4. The experiment conducted for 24 h, at 37 °C, under continuous stirring (50 rpm), using a 708-DS dissolution apparatus (Agilent, Santa Clara, CA, USA).

To determine the Ibuprofen release kinetics, 500 μL of supernatant were automatically collected at predetermined time points using an autosampling station (850-DS, Sampling Station, Agilent, Santa Clara, CA, USA) and replaced with an equal volume of fresh PBS. The collected samples were analyzed for Ibuprofen content by UV-Vis spectroscopy (NanoDrop One, Thermo Scientific). Ibuprofen concentrations were determined using a calibration curve prepared from standard Ibuprofen solutions in PBS (pH 7.4). Samples were diluted with PBS when necessary to ensure that their absorbance values were within the linear range of the calibration curve. The cumulative percentage of Ibuprofen released at each time point was calculated based on the amount of drug measured in the collected samples relative to the total amount of Ibuprofen initially loaded into microparticles [34]. The cumulative release data were then plotted against time to obtain the Ibuprofen release profiles and evaluate the release kinetics.

#### 2.3.9. Theoretical Analysis of Drug Release

The Korsmeyer–Peppas model is particularly useful for investigating drug release from polymeric delivery systems when more than one mechanism, such as diffusion, polymer relaxation, and matrix swelling, may contribute to the release process.

The Korsmeyer–Peppas (also known as the Ritger–Peppas) model was applied to further investigate the drug release mechanism from the prepared microparticles. The relationship between the fraction of drug released and release time is described by the following equation:

(2) $$\frac{M_{t}}{M_{\infty }}={kt}^{n}$$

where Mt = mass of drug released at time t, M∞ = mass of drug released as t approaches infinity, k = transport constant. The release exponent n provides information about the possible drug-release mechanisms at play.

#### 2.3.10. Haemolysis Assessment

Haemolysis testing represents a mandatory requirement for materials that come into direct contact with blood. Hemolysis is the destruction of red blood cells, resulting in the release of hemoglobin and other components into the surrounding fluid. If this destruction is significant, it can lead to dangerous pathological conditions. To assess the hemocompatibility of the obtained microparticles, the spectrophotometric method was used [37,38]. The tests were started only after the institutional ethical clearance and the corresponding informed consent were signed. The human blood sample used was collected in 3.2% sodium citrate vacutainer tubes from a healthy non-smoking volunteer. The collected blood (4 mL) was centrifuged at 2000 rpm for 5 min to remove the plasma, and the sedimented red blood cell (RBC) suspension was washed several times with saline solution purchased from Fresenius Kabi (Bad Homburg, Germany). The RBC suspension was finally diluted with saline solution to a final volume of 20 mL. Then, 0.5 mL of the microparticles suspension in normal saline were added over 0.5 mL of the RBC suspension, obtaining final concentrations of 25 and 50 µg particles/mL. To prepare the positive (100% lysis) and negative (0% lysis) control samples, 0.5 mL of 2% Triton X 100 and saline, respectively, were added over the RBC suspension. The samples were then incubated in an oven at 37 °C for 1.5 and 3 h. After this periods, the samples were centrifuged for 5 min at 2000 rpm. The absorbance of oxyhemoglobin in the supernatant was measured at 540 nm using a NanoDrop spectrophotometer. The hemolysis degree was calculated using the Equation (3):

*Hemolysis*% = (*Abs_Sample_* − *Abs_Negative control_*)/(*Abs_Positive control_* − *Abs_Negative Control_*) (3)

The experiments were performed in triplicate.

#### 2.3.11. Effect of Microparticles on Cell Viability

The cell viability was assessed by the 3-(4,5-dimethylthiazol-2-yl)-2,5-diphenyltetrazolium bromide (MTT) assay using adult human dermal fibroblasts and a cell line (HDFa). The experiment comprised the following sequential stages: preparation of the nutrient medium necessary for cell proliferation; thawing of the cell line and cultivation of dermal-derived fibroblast cells (HDFa) in culture vessels (Nunc™ EasYFlask™ of 25 cm^2^ with a working volume of 7 mL); change of nutrient medium; microscopic analysis using an inverted microscope (CKX41, OLYMPUS (Tokyo, Japan) with image acquisition system (camera) and QuicKPHOTO Camera 3.0 software); passage and detachment of the cell monolayer with trypsin EDTA 0.05% and obtaining subcultures; counting cells using the automatic cell counter (EVE™ Automatic cell counter with EVE PC software, Version 1.0.3) and evaluating cell viability using the exclusion test with the vital dye trypan blue 0.4%; cultivation of cells from the subculture in 96-well plates; the placing materials in direct contact with fibroblast cells; determination of cell viability after 24 and 48 h of incubation by the colorimetric method with MTT. The samples were read spectrophotometrically at a wavelength of 570 nm using the Multiskan FC automated microplate reader and the Skanlt Software 4.1 software.

#### 2.3.12. Antimicrobial Assay

The antimicrobial activity testing was performed by the disk diffusion method (diffusimetric method), using micro-tablets impregnated with a known quantity of the particle suspension. As culture media, we used Columbia Agar supplemented with 5% sheep blood for the growing of reference strains (*Streptococcus pneumoniae* ATCC 49619). The media used for antimicrobial susceptibility testing (AST) was Mueller-Hinton Fastidious (MHF) with horse blood.

After preliminary tests, the concentration that produced a measurable zone of inhibition (40 µg microparticles/1 µL distilled water) was established. The suspension thus obtained was well dispersed by vortexing to ensure a uniform particle distribution in the aqueous environment. Immediately, 10 µL of each compound were taken and impregnated onto sterile filter paper disks (micro-tablets). The samples used for impregnation of the micro-tablets were the following: (i) MPs-4 microparticles that were used as a control sample; (ii) ZnONPs; (iii) MPsZO-3 microparticles with a lower amount of ZnO nanoparticles; (iv) MPsZO-4 microparticles with a higher amount of ZnO nanoparticles (according to Table 1). Micro-tablets containing the suspensions of microparticles were transferred onto the surface of the Mueller-Hinton medium, which has been previously confluently inoculated with the reference strain *Streptococcus pneumoniae* ATCC 49619. An inoculum of 106 CFU was prepared from the reference culture and inoculated onto the agar surface by discharging the swab moistened with the inoculum. It was left for 15 min for absorption and fixation in the medium. The plates were incubated for 24 h at 37 °C. To ensure the reproducibility of the results, the tests were replicated five times. The diameter of the growth inhibition zones for the strain *Streptococcus pneumoniae* ATCC 49619 was measured using a graduated ruler.

## 3. Results and Discussion

A key innovation of the developed system is the synergistic integration of ZnONPs and Ibuprofen within a biopolymeric matrix. This multifunctional platform, capable of exerting antimicrobial effects while concurrently mitigating inflammation, can address both the source and the effects of infection. Firstly, these microparticles were characterized from a physicochemical point of view by different techniques. Then, their in vitro biological evaluation was carried out in order to assess their potential use in biomedical applications.

### 3.1. Structural Characterization

The FTIR spectra for CMCS, ZnONPs and for microparticles with and without ZnONPs are presented in Figure 2 and Figure 3 and in Table 2 and Table 3.

Table 2 and Figure 2 present the FTIR analysis results for CMCS and the ZnO-free microparticles (MPs-2, MPs-3, and MPs-4). Obtained results confirmed the interaction between PVA and CMCS and demonstrated the preservation of the characteristic chemical structure of CMCS following microparticle formation. The broad bands observed at approximately 3300 cm^−1^ can be attributed to the stretching vibrations of O–H groups in PVA and CMCS, as well as hydrogen-bonded water molecules. The bands at 2931 and 2887 cm^−1^ are attributed to the asymmetric and symmetric stretching vibrations of aliphatic C–H groups, respectively. The appearance of new signals around 1650 and 1545 cm^−1^, which may be associated with carbonyl and amide vibrations, confirm the reaction between the amine functional groups of CMCS and hydroxyl groups of PVA with the carboxylic groups of CMCS. The presence of peaks at 1467 cm^−1^ in the spectrum of the microparticles samples confirms the esterification reaction that occurred between the hydroxyl groups of PVA and the carboxyl groups of CMCS. The disappearance, shift, or amplification of some peaks in the CMCS spectra and the appearance of new signals in the microparticle spectrum confirm the formation of new bonds. The strong band situated at approximately 1051 cm^−1^ is particularly characteristic for the polysaccharide/PVA matrix and can be attributed to the C–O and C–O–C stretching vibrations. Peaks in the 685–711 cm^−1^ region present in the spectra of all analyzed samples can indicate skeletal vibrations or other structural features in the polymer matrix, including potential interactions between the two polymers [39,40].

FTIR spectra of ZnONPs and microparticles in the absence and presence of ZnONPs (MPs-4 and MPsZO-4) in Figure 3. The main absorption bands and their assignments are summarized in Table 2. From this figure, it can be noticed that the characteristic peaks of the ZnONPs, observed at 428 cm^−1^, 697 cm^−1^, 832 cm^−1^, 1047 cm^−1^, 1389 cm^−1^, and 1501 cm^−1^, are found in the biocomposite microparticle samples [41]. The broad absorption band is found at 3416 cm^−1^, which is attributed to surface hydroxyl groups. The bands at 1501 cm^−1^ can be attributed to adsorbed water and peak at 1389 cm^−1^, indicating the O–H bending as carboxylic acid. The peak at 1047 cm^−1^ is attributed to the stretching vibration of the C-N bond of the primary amine or to the stretching vibration of the C-O bond. Peak from 832 cm^−1^ indicates the existence of functional groups associated with the C=C bending. The peaks in the region between 700 cm^−1^ and 400 cm^−1^ are assigned to the Zn–O bond. Therefore, the band at 428 cm^−1^ corresponds to the metal–oxygen (ZnO stretching vibrations) vibration mode. For MPS-ZnO-4 microparticles, the FTIR spectrum contains characteristic bands corresponding to the CMCS/PVA polymer matrix, as well as the incorporated ZnONPs. The broad peak at 3376 cm^−1^ and 3289 cm^−1^ indicates the presence of abundant hydroxyl groups and hydrogen bonding interactions within the polymer matrix. The presence of peaks at 1589 cm^−1^, 1399 cm^−1^, 1319 cm^−1^, 1057 cm^−1^, and 830 cm^−1^ in the spectrum of the MPS-ZnO-4 sample can be attributed to the stretching vibrations of the carboxylate groups COO^−^; C–O; C–C; C–O–C in the CMCS/PVA polymer matrix. It is important to note that the presence of peaks at 494 cm^−1^, 426 cm^−1^, and 417 cm^−1^, corresponding to Zn–O vibrations (as seen from peak deconvolution) [42], provides direct spectroscopic evidence for the incorporation of ZnONPs into the CMCS/PVA microparticle matrix. Therefore, the FTIR spectra demonstrate on the one hand the interaction between the two polymers and, on the other hand, the presence of ZnONPs in the polymer matrix.

### 3.2. Microparticles’ Mean Diameter

The mean diameter of the microparticles in the absence and in the presence of ZnONPs was analyzed in acetone to evaluate the size of the microparticles in the unswollen state but also in different aqueous solutions such as: ultrapure water, physiological saline solution, PBS (pH = 7.4), and FBS 10%, which mimic the biological environment. The obtained results are presented in Table 3 and Figure 4.

All analyzed microparticles present a monomodal size distribution, except for samples containing ZnONPs, and a polydispersity ranging between 0.33 and 0.59. Comparing the average diameters of all analyzed microparticles, it was observed that the introduction of ZnONPs into the system led to a slight increase in their diameter, which was expected. This increase can be related to the penetration of ZnONPs into the meshes of the polymer network, thus leading to a diameter increase. Microparticles without ZnONPs, analyzed in acetone, had mean diameters ranging between 533 nm and 943 nm, whereas the average sizes in the presence of ZnONPs varied between 726 nm and 1054 nm. The obtained results (Table 2 and Figure 4) revealed that with the increase of PVA amount in the microparticles composition, the average diameter decreased, with the exception of the MPs-5 sample. In the case of microparticles containing only CMCS, only intramolecular amide bonds are formed between the amine and carboxyl groups on the chitosan chains. Consequently, the cross-linking density is low and the diameter of the microparticles is higher as the macromolecular chains are in an extended state. When PVA was added to the system, the cross-linking density increased as a result of the esterification reaction between the hydroxyl groups on the PVA chains with the carboxyl groups on the CMCS chains. Consequently, the diameter of the microparticles decreases due to a denser and more compact network [43]. It is also known that PVA is a macromolecular surfactant that can increase emulsion stability and, therefore, lead to a decrease in particle size [44,45]. In the case of the MPs-5 sample, uncross-linked PVA chains remain in the polymer network, thus leading to an increase in the diameter of the microparticles.

The MPsZO-1 sample was chosen to be analyzed in four different media, namely: ultrapure water, saline solution, PBS (pH = 7.4), and FBS 10% (Figure 4C). Ultrapure water and saline solution were chosen because they are usually used as a medium for intravenous injection of drugs. PBS (pH = 7.4) and FBS 10% solutions were used as they mimic the physiological environment. From Figure 4C, it was observed that the microparticles had the smallest diameter in ultrapure water. In FBS 10%, the diameter was close to that found in ultrapure water, which reveals a good stability of the microparticles in this environment. This behavior may be due to the proteins from FBS, which can stabilize the microparticles. The increase in particle size observed in saline solution can be attributed to the hydration and swelling of the hydrophilic PVA/CMCS network, together with ion-induced relaxation of polymer chains. The presence of electrolytes promotes water uptake and expansion of the polymer matrix, resulting in an increased hydrodynamic diameter.

Statistical analysis revealed significant differences in microparticle diameter among samples without and with ZnONPs. For the microparticles without ZnONPs (MPs-1–MPs-5), one-way ANOVA demonstrated a significant effect of particles composition on mean diameter (F(4,10) = 40.49, *p* < 0.0001 (*)). The mean diameter ranged from 547.0 ± 17.8 nm for MPs-4 to 961.7 ± 32.3 nm for MPs-2. Tukey’s HSD post-hoc analysis confirmed significant differences between selected microparticles, indicating that changes in PVA and DMT-MM content were associated with substantial changes in particles size.

A similarly significant effect was observed among the microparticles containing ZnONPs (MPsZO-1–MPsZO-4; F(3,8) = 86.74, *p* < 0.0001 (*)). The particle diameter decreased from 1121.3 ± 58.9 nm in MPsZO-1 to 713.7 ± 21.4 nm in MPsZO-4. Tukey’s HSD analysis demonstrated significant differences among the MPsZO microparticles, with MPsZO-1 and MPsZO-2 exhibiting significantly larger particles than MPsZO-3 and MPsZO-4. These findings indicate that changes in samples composition significantly influence the mean diameter of the prepared microparticles.

### 3.3. Zeta Potential of Microparticles

Zeta potential measurements were carried out in both PBS (pH = 7.4) and FBS 10% to evaluate their stability in an environment that mimics physiological conditions but also in 0.9% saline solution. It is well known that particulate systems that have zeta potential values lower than −25 mV and higher than +25 mV are recognized as stable [46]. The obtained results are presented in Table 4 and showed that the highest values are obtained in PBS (pH = 7.4). The zeta potential values in physiological serum ranged between −13.3 mV and −20 mV. In PBS solution (pH = 7.4), it was between −19.6 mV and −31 mV, and in 10% FBS solution, it ranged between −8 mV and −18.5 mV. As a general remark, it is found that with the increase in PVA amount in the system, the zeta potential values decrease due to an increase probability of the esterification reaction between CMCS and PVA. Thus, there will be fewer carboxyl groups available, which, in the slightly alkaline environment, are transformed into carboxylate groups, leadingto a decrease in the zeta potential value. These results are in accordance with those obtained in the dimensional analysis. On the other hand, the introduction of ZnONPs in the system led to a decrease in the zeta potential value.

### 3.4. Morphological Characterization

#### 3.4.1. TEM Microscopy

Figure 5 shows the TEM microscopy for microparticles in the absence of ZnONPs (MPs-2 and MPs-4) and the MPsZO-3 type microparticles containing the highest amount of ZnONPs. All three types of microparticles are spherical in shape and the sizes range from approximately 500 nm to 1 µm. Also, TEM images revealed a uniform morphology without clearly distinct phase-separated domains, suggesting good integration of CMCS and PVA into the cross-linked polymer network. The results obtained are in agreement with those obtained from DLS analysis. The MPs-2 microparticles containing only CMCS have dimensions of approximately 900–950 nm, while the MPs-4 microparticles containing both CMCS and PVA have dimensions of approximately 500 nm. As explained above, the addition of PVA to the system led to an increase in the cross-linking density and implicitly to a decrease in the dimensions of the microparticles. PVA also acts as a surfactant, which improves the stability of the emulsion and, consequently, reduces the particle size in diameter. The inclusion of ZnONPs in the system led, as expected, to an increase in the diameter of the microparticles. The MPsZO-3 sample containing the highest amount of ZnO shows diameters of approximately 800 nm. The presence of several populations of microparticles can also be observed, as evidenced by the DLS analysis, which may indicate the presence of ZnONPs in the system.

#### 3.4.2. SEM-EDAX Analysis

The elemental composition of the microparticles in the absence (MPs-4) and in the presence of ZnONPs (MPsZO-1, MPsZO-2, MPsZO-3) was investigated by SEM-EDAX analysis. The obtained results, that are presented in Figure 6, confirmed the presence of characteristic peaks for carbon (C), oxygen (O), and nitrogen (N) in all analyzed samples, as expected.

The EDAX spectra of the MPsZO samples confirmed the presence of ZnONPs embedded in the polymeric matrix of the microparticles. Approximately 85% of the initial amount of ZnONPs was incorporated into the polymeric microparticles. Furthermore, increasing the amount of ZnONPs in the system led to an increase in the intensity of the Zn characteristic peak, as expected.

### 3.5. Microparticles Behavior in Aqueous Solutions

The evaluation of the swelling degree is a test preceding the release test and was carried out to evaluate the behavior of the microparticles in a fluid that simulates the biological environment. Microparticles in the absence and in the presence of ZnONPs were analyzed in PBS solution (pH = 7.4), at a temperature of 37 °C, and the obtained results are presented in Figure 7. The swelling degree of the microparticles without ZnONPs after 24 h varied between 1620% and 2055%, being influenced by the ratio between the two polymers (Figure 7A). The obtained results revealed that with the increase of the PVA amount in the composition of the microparticles, the swelling degree decreased due to a higher cross-linking density. The samples containing only CMCS (MPs-1 and MPs-2) have a higher amount of free carboxyl groups than those containing PVA (MPs-3, MPs-4, and MPs-5). In the slightly alkaline environment, these groups are ionized to carboxylate groups, which will lead to electrostatic repulsions and thus increase the swelling degree. With the addition of PVA to the system, both the amidation reaction between the amine groups and the carboxyl groups, as well as the esterification reaction between the hydroxyl groups and the carboxyl groups, take place, causing an increase in the cross-linking density and, consequently, a decrease of the swelling degree.

The swelling degree of the microparticles containing ZnONPs varied between 650% and 1550% (Figure 7B). The inclusion of ZnONPs in the microparticles led to a significant decrease in the swelling degree, as expected. ZnONPs were present in the pores of the polymer network, and therefore the available volume for the penetrability of the aqueous solution through the polymer membrane of the microparticles is limited, leading finally to a decrease in water uptake. These results are in good agreement with the dimensional analysis presented above and with the literature data. For example, Azimi et al. developed a new hydrogel loaded with insulin that responds to pH and temperature changes. The authors found that the swelling of the hydrogel is significantly influenced by both pH and temperature. In fact, increasing the pH led to increased swelling due to the electrostatic repulsion between the sulfonate groups (SO^3−^) [47]. A different behavior was reported by Amri et al. for chitosan/PVA/montmorillonite hybrid polymer systems. In that study, increasing the PVA content enhanced the swelling capacity, which was attributed to the hydrophilic character of PVA, the large number of hydroxyl groups, increased water affinity, and polymer-chain relaxation. In contrast, increasing the PVA content in the present CMCS/PVA system resulted in a decrease in the swelling degree. This difference may be related to the different chemical nature of the chitosan-based polymer used in the two systems. Unlike native chitosan, CMCS contains additional carboxylic groups, which increase the ionic character of the polymeric matrix and can substantially contribute to water uptake through ionization and electrostatic repulsion. In the present study, increasing the PVA content may favor stronger intermolecular interactions and a more compact polymeric network, partially counteracting the intrinsic hydrophilicity of PVA and limiting the expansion of the matrix. An additional difference between the present system and the previously reported chitosan/PVA-based materials is the incorporation of ZnO nanoparticles. In the present study, ZnO incorporation markedly decreased the swelling degree from 1620–2055%; for the ZnO-free microparticles, to 650–1550% for the ZnO-containing formulations. This behavior indicates that ZnO can act as a structural modulator of the polymeric matrix, restricting water penetration and polymer-chain expansion. From a formulation perspective, this may represent an advantage because it allows the excessive swelling of the highly hydrophilic CMCS-based matrix to be easily modulated and provides an additional means of adjusting the hydration and structural stability of the microparticles. However, excessive restriction of water uptake may also represent a limitation, as reduced hydration could affect the diffusion and subsequent release of incorporated active substances. Therefore, the present CMCS/PVA/ZnO system offers the advantage of tunable swelling behavior through modification of both the polymer ratio and ZnO content, although optimization of these parameters is necessary to achieve an appropriate balance between water uptake, structural stability, and drug release [48].

### 3.6. Microparticles Stability Studies

The stability of the obtained microparticles in PBS medium (pH = 7.4) was evaluated after 1 week of storage at 37 °C. Therefore, the results of the degradation degree (Δm%) of the microparticles in the absence and in the presence of ZnONPs at different ratios between the two polymers are presented in Figure 8.

Thus, the percentage of degradation of the microparticles after 7 days was between 14% and 40%. It was found that the highest degree of degradation (40%) was observed for MPs-1 sample, which has only CMCS in its composition, and the molar ratio between the COOH groups from CMCS and DMT-MM was 2/1. In contrast, the MPs-2 sample, for which the same amount of CMCS was used but with a molar ratio between the COOH groups from CMCS and DMT-MM of 3/1, had a degradation degree of approximately 33.5%. This result reveals that increasing the amount of activator improved the reaction between the amine and carboxyl groups, forming a better and more stable cross-linked network in the physiological environment. Moreover, the addition of PVA to the microparticle composition led to a decrease in the degradation degree. It was observed that with the increase in the amount of PVA, the degree of degradation decreased to 17.5%. This phenomenon is due to the appearance of ester groups that lead to an increase in the cross-linking density, and, consequently, to an increase in the stability of the microparticles in the aqueous environment. The microparticles containing ZnONPs showed the best stability, as expected. Their degradation percentage varied between 14% and 29% and decreased with increasing ZnONPs content in their composition. As explained in the swelling test, the ability of the aqueous solution to penetrate the pores of the polymer network is lower because ZnONPs occupy part of the free volume of meshes within the polymer network. Consequently, the degradation percentage of these particles is lower and their stability is improved.

### 3.7. Loading of Microparticles with Ibuprofen

Ibuprofen was used as a model anti-inflammatory drug. For the loading test, both microparticles in the absence (MPs-2, MPs-3, MPs-4) and in the presence of ZnONPs (MPsZO-1) were used. The loading degree varied between 35% and 39% and the results are presented in Figure 9. The highest loading degree was found in the MPs-2 sample, the sample that has only CMCS in its composition and therefore the lowest cross-linking density and the highest swelling degree. In general, the results obtained correlate with the DLS analysis and the swelling degree.

### 3.8. In Vitro Release Studies of Ibuprofen

Drug release from polymer matrices is a complex process. Achieving controlled release is essential to reduce the need for frequent dosing and minimize adverse effects associated with prolonged used of NSAIDs, such as nausea and internal bleeding reported for commonly used drugs. Ibuprofen, despite its well-established anti-inflammatory, analgesic, and antipyretic efficacy, presents a short plasma half life of approximately 2 h, which necessitates repeated dosing to maintain therapeutic concentrations. Figure 10 shows the release curves in the PBS solution (pH = 7.4) for: (i) free Ibuprofen (Ibu); (ii) Ibuprofen loaded into microparticles in the absence of ZnONPs (MPs-2, MPs-3, MPs-4); (iii) Ibuprofen loaded into microparticles with ZnONPs (MPsZO-2). In the case of free drug, a linear and total release was observed after approximately 3 h from the start of the experiment. The release degree of Ibuprofen from microparticles varied between 84% and 100% and occurred in two stages. In the first 3 h, a faster release of the drug occurs, which may be due to a burst release of drug molecules adsorbed on the surface of microparticles. This initial burst effect is a well-documented phenomenon in polymeric drug delivery systems and has been reported for chitosan-based microparticles loaded with NSAIDs, where surface-adsorbed drug fractions are rapidly released upon contact with the dissolution medium. A biphasic release profile, characterized by an initial rapid phase followed by a slower sustained phase, has similarly been described for NSAID-loaded chitosan microspheres in PBS at pH 7.4, a pattern consistent with the results obtained in the present study. In the second stage, a slower drug release was observed until equilibrium. In this case, the drug was most likely dispersed in the membrane but also in the meshes of the polymer network of the microparticles. This diffusion-controlled second phase reflects the gradual permeation of the dissolution medium through the swollen polymer matrix, followed by the slow outward diffusion of drug molecules entrapped within the cross-linked network. Ths behaviour is frequently reported for hydrophilic polymer-based particulate systems. Composite microparticles incorporating inorganic nanoparticles have been shown to exhibit prolonged drug release and a reduced burst effect compared to simpler microparticulate formulations. This is in accordance with the slightly decreased release degree observed for ZnONP-loaded formulations in the present study. For the MPs-2 sample, a 100% release was observed. It was noted that the introduction of PVA into the system led to a decrease of the release degree. These results correlate with the DLS analysis, with the degree of swelling but also with the evolution of the degree of loading.

### 3.9. Theoretical Analysis of Drug Release

The experimental release data were fitted to the Korsmeyer–Peppas model using the initial 60% of the cumulative drug release data, as recommended for assessing the predominant drug transport mechanism. The release exponent (n) was used to characterize the release mechanism, and the fitting results are presented in Table 5. For the investigated microparticulate systems, the obtained (n) values suggest that drug release is predominantly controlled by diffusion through the polymeric matrix. In particular, (n) values within the Fickian diffusion range indicate that the release process is mainly governed by molecular diffusion driven by the concentration gradient. Therefore, the release mechanism can be characterized as a predominantly Fickian diffusion. The higher value of the kinetic constant (k) observed for plain Ibuprofen indicates a comparatively faster apparent release rate than that observed for the microparticulate formulations. This finding is consistent with the absence of the polymeric matrix, which otherwise acts as a diffusion barrier and can retard drug release. R^2^ values approaching 1 indicate a strong agreement between the experimental release profile and the model, supporting the suitability of the Korsmeyer–Peppas equation for describing the release behavior of the investigated formulations.

### 3.10. Hemolysis Test

Another in vitro test used to evaluate the biocompatibility of the microparticles was hemolysis, given that these systems are intended as carriers for active principles and will probably reach the circulatory system. This analysis was carried out by the spectrophotometric method and the results obtained are presented in Figure 11. The experiments were performed in triplicate, and error bars represent the standard deviation.

The results obtained from this evaluation suggest that the MPs and MPsZO microparticles have good compatibility with the blood environment, as they do not produce a degree of hemolysis greater than 3% [49]. Another observation that can be drawn from Figure 11 is that there is a slight increase in the hemolytic percentage with increasing microparticle concentration for all tested samples. Furthermore, the influence of exposure time on the erythrocyte lysis degree was evident, after 180 min being higher than after 90 min. However, the mixture of polymers in the composition of the MPs microparticles did not show a significant influence, whereas the presence of ZnONPs induced some changes in the percentages of hemolysis. In fact, increasing the amount of ZnONPs in the MPsZO composition leads to an increased lysis degree.

### 3.11. Cytotoxicity Test

Adult dermal fibroblasts are mesenchymal cells that constitute the stroma across tissues and play an essential role in the regeneration and maintenance of the skin structure [50,51]. Cytotoxicity assay can represent a relevant cellular model for assessing the safety of materials intended for contact with cells because it allows the identification of adverse effects of the tested substances or materials on cellular viability, morphology, and function. This behaviour can contribute to the estimation of biocompatibility and the prevention of potential toxic or inflammatory reactions before clinical application. In this context, microparticles without (MPs-4) and with ZnONPs (MPsZO-3 and MPSZO-4) at different concentrations (25 µg/mL; 50 µg/mL and 100 µg/mL) were placed in direct contact with fibroblasts cells for 24 and 48 h and the cytotoxicity was evaluated using the standard MTT method. The results allowed the evaluation of the dose-dependent effects of the analyzed materials and provided essential information regarding their safety and limit of use in contact with human cells. The obtained results that are presented in Figure 12 showed that most samples analyzed at both 24 and 48 h are non-cytotoxic. All experiments were performed in triplicate, and in Figure 12, the error bars represent the standard deviation.

After 24 h of exposure of cells to microparticles, the degree of cell viability varied between 94.22% and 80.15%. After 48 h of treatment, cell viability decreased to 91.92% and 72%, indicating that they remained in the non-cytotoxicity zone, with the exception of the MPSZO-3 and MPSZO-4 samples, which, at a concentration of 100 µg/mL and after 48 h of treatment, showed a cell viability of 72% and 74%, respectively, falling within the range of weak cytotoxicity. As expected, with increasing microparticle concentration, the degree of cell viability decreased.

### 3.12. Antimicrobial Assay

Antimicrobial resistance is a major global problem that leads to difficulties in treating infections and, therefore, the identification of new antimicrobial agents is necessary. The methods frequently used to investigate the antimicrobial effect are disk diffusion and broth dilution, whereas more advanced techniques offer superior sensitivity and throughput, albeit at a higher cost [52]. Antimicrobial microparticles/nanoparticles containing silver or zinc oxide nanoparticles exhibit strong antimicrobial activity against bacteria, fungi, and viruses due to their ability to disrupt bacterial cell membrane walls and damage intracellular structures [53]. Regarding antiviral activity, metal and metal oxide nanoparticles can interact with surface components of viral particles, such as spike glycoproteins, thereby interfering with viral attachment, entry, and uncoating in host cells. In addition, the generation of reactive oxygen species (ROS) can induce oxidative damage to viral macromolecules, including nucleic acids, capsid proteins, and lipid envelopes. Nanoparticles may also interact with viral glycoproteins and disrupt disulfide bonds, leading to structural alterations and loss of viral infectivity [54]. The results presented in Figure 13 show that ZnONPs and biocomposite microparticles of the MPsZO-4 type have a strong activity against *Streptococcus pneumoniae* strain.

Two types of microparticles with the same molar ratio between polymers but different amounts of ZnONPs were tested (namely MPsZO-3 with a lower content of ZnONPs and MPsZO-4 with a higher content of ZnONPs). The ZnONPs samples showed good antimicrobial activity against the *Streptococcus pneumoniae* strain at a concentration of 40 µg/µL with an inhibition zone diameter of approximately 14 mm. It was found that the MPsZO-3-type microparticles did not show an antimicrobial effect. On the other hand, the MPsZO-4 sample showed good antimicrobial activity with an inhibition zone diameter of approximately 16 mm. Control MPs samples prepared under identical conditions, but without Ibuprofen and ZnONPs, showed no detectable antibacterial activity, indicating that the preparation procedure, including the use of DL-limonene as the oil phase, did not contribute to the observed antimicrobial effects of the ZnONP-loaded microparticles.

The antibacterial activity of ZnONP-loaded microparticles is attributed to the presence of ZnO, which may exert their antimicrobial effect through several complementary mechanisms, including ROS generation, Zn^2+^ ion release, and direct interaction with the bacterial cell envelope [55,56]. These processes may promote oxidative stress and membrane damage, leading to inhibition of bacterial growth. The absence of antibacterial activity for MPsZO-3 and the clear inhibition observed for MPsZO-4 suggest that the antimicrobial response is dependent on the amount of ZnONPs incorporated into the microparticles. The absence of activity in the unloaded MPs control further confirms that the antibacterial effect is associated with ZnONPs rather than the polymer matrix or the DL-limonene-based preparation process.

## 4. Conclusions

Microparticles based on CMCS and PVA were successfully obtained, using a simple water/oil emulsion method and a non-toxic activating agent, capable of generating ester and amide bonds between the two polymers. The formation of specific bonds between the two polymers, as well as the incorporation of ZnONPs, was efficiently achieved, as confirmed by FTIR spectroscopy. Morphological characterization showed that the shape of the microparticles was spherical, and their size was controlled by the composition. Moreover, the microparticles with ZnONPs showed slightly increased dimensions. Studies on the swelling degree and stability in aqueous media demonstrated that the obtained microparticles present physicochemical properties suitable for biomedical applications. In addition, the in vitro biological evaluation indicated suitable hemocompatibility, cytocompatibility, and antibacterial activity, suggesting that these microparticles can be successfully used for the development of controlled drug delivery systems. The obtained results confirm the potential of these microparticles as carriers for controlled delivery of Ibuprofen and ZnONPs, particularly in the current context where alternative strategies to combat microbial infections and antibiotic resistance are actively being sought. The incorporation of ZnONPs was intended to confer antimicrobial properties to the system, while the co-loading of Ibuprofen was designed to provide anti-inflammatory activity, thereby addressing both the infectious cause and the associated inflammatory response. Future investigations will therefore encompass anti-inflammatory testing and in vivo studies to further validate the therapeutic potential of these systems for pulmonary administration.

## Figures and Tables

**Figure 1:**
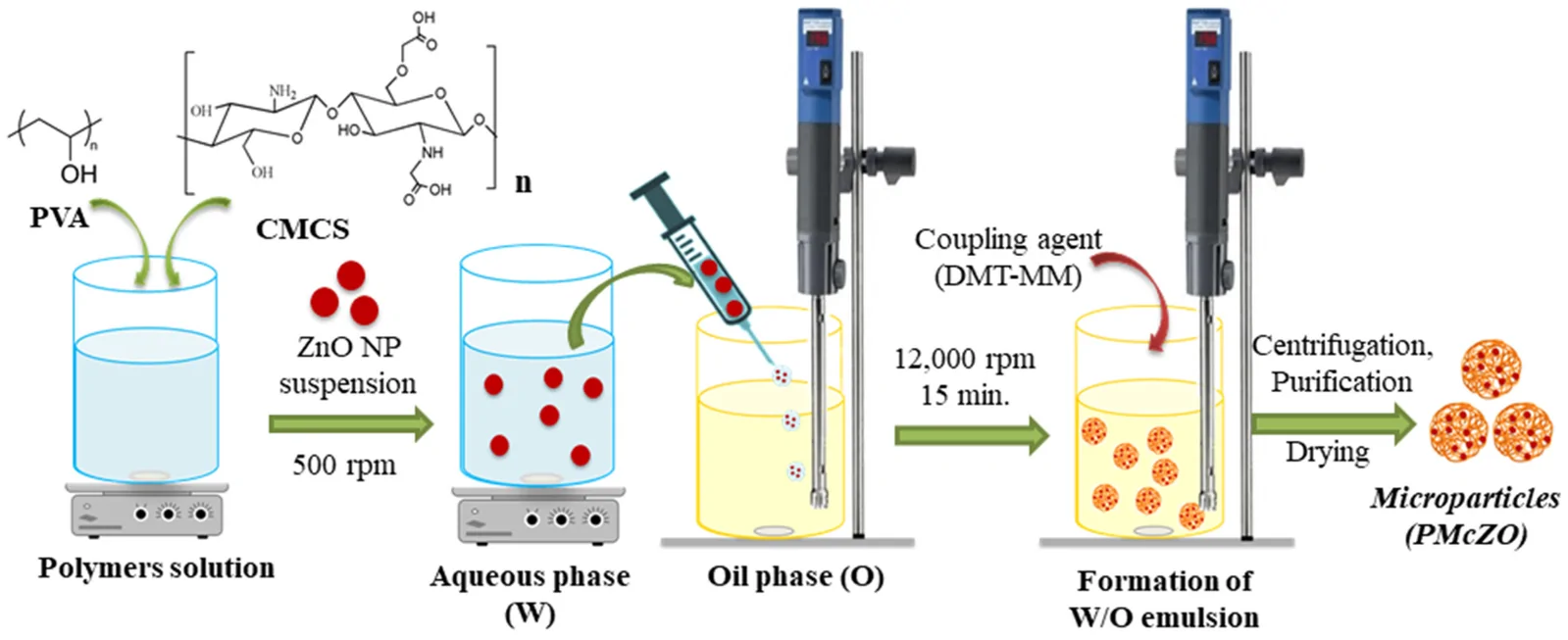
Schematic illustration of the microparticle synthesis protocol.

**Figure 2:**
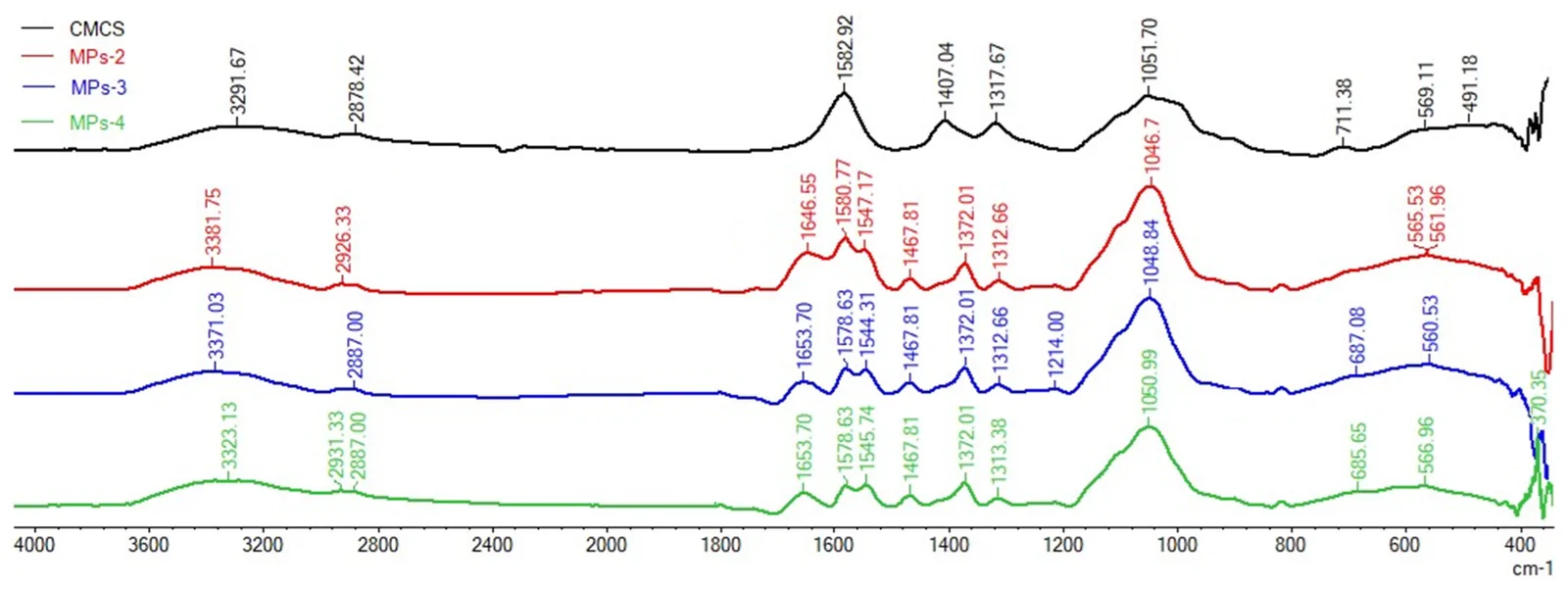
FTIR spectra for CMCS and microparticles without ZnONPs.

**Figure 3:**
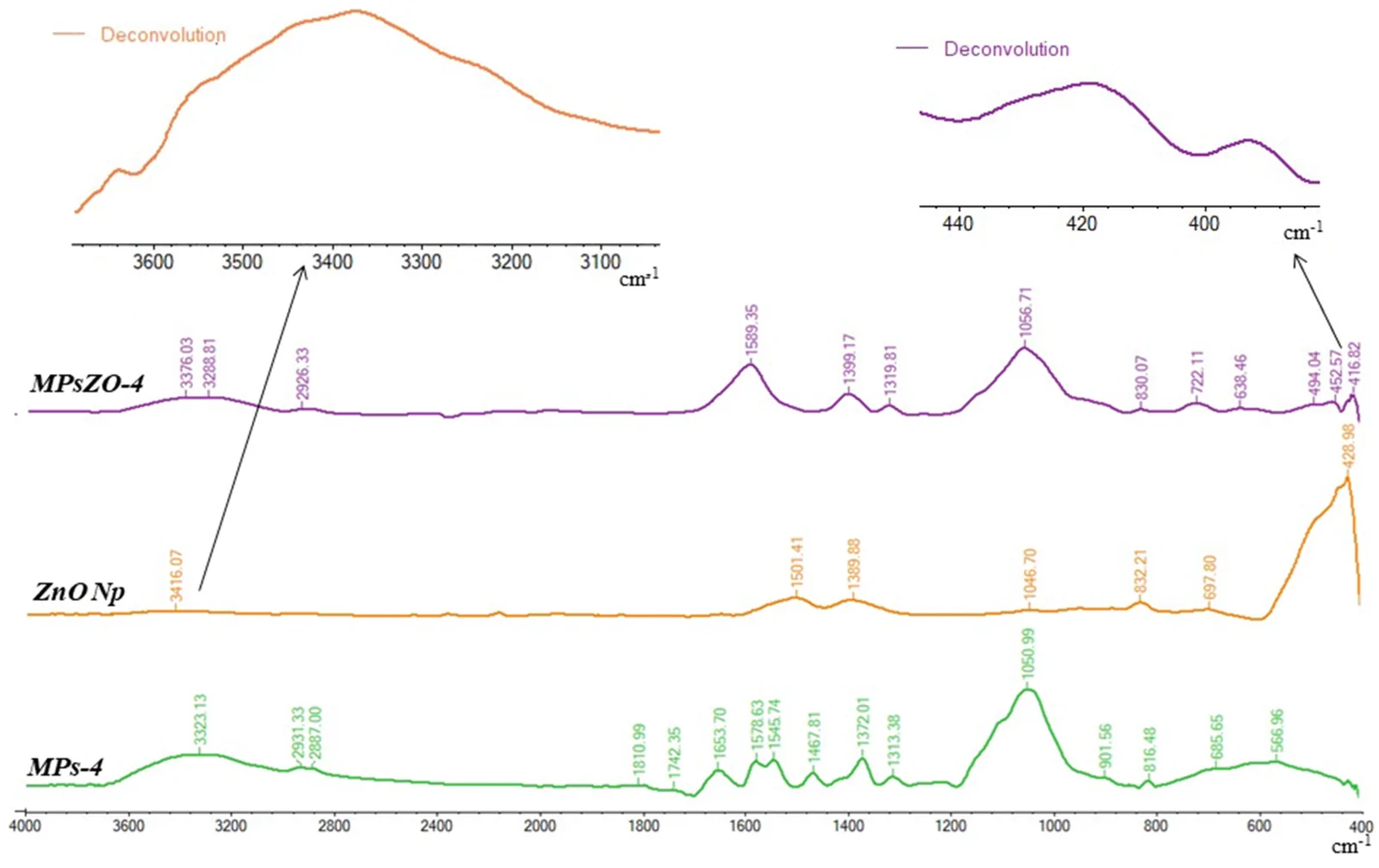
FTIR spectra for ZnONPs and microparticles containing ZnONPs.

**Figure 4:**
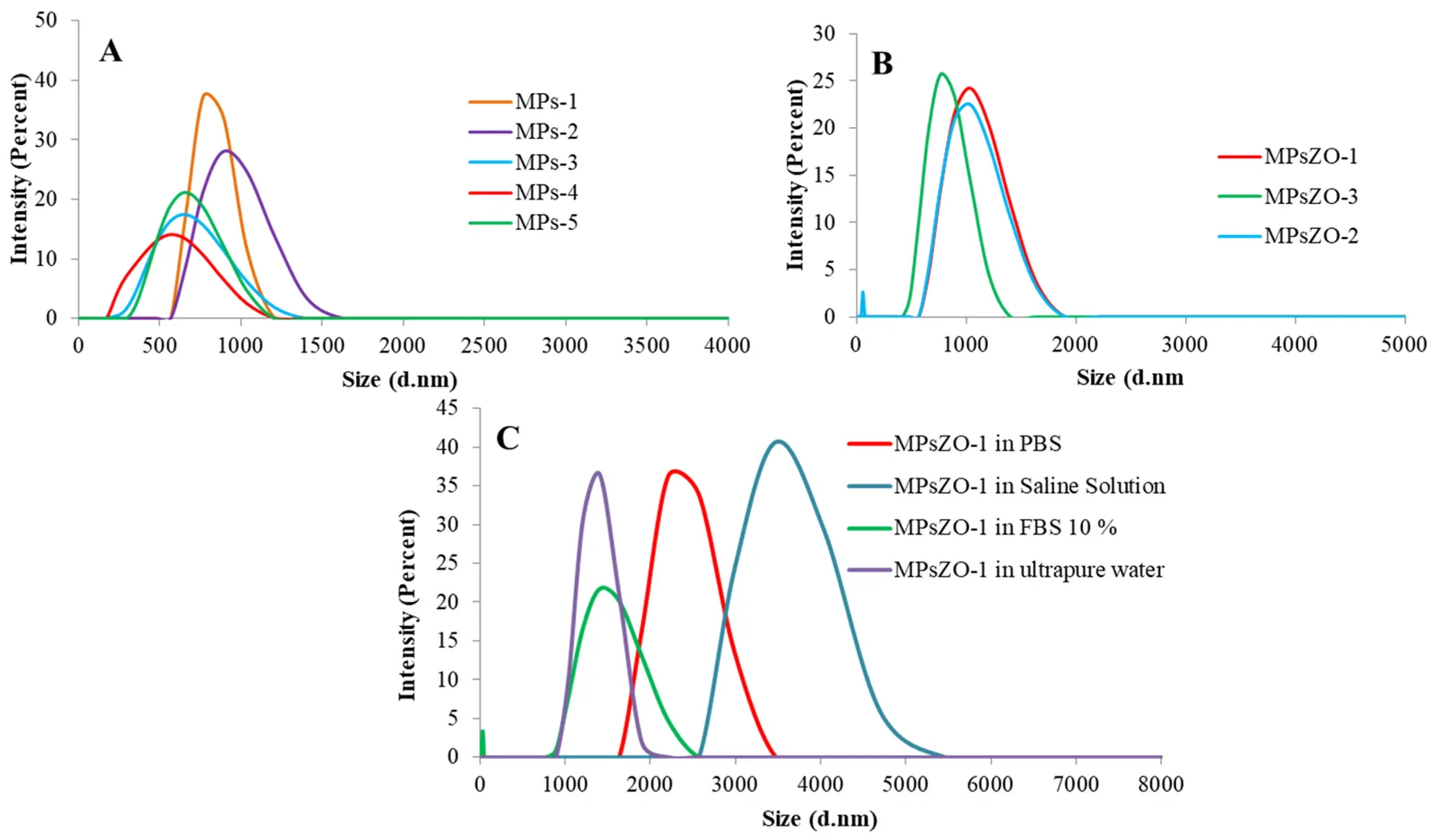
Granulometric distribution of: (**A**) microparticles without ZnONPs at different ratios between polymers; (**B**) microparticles containing different amounts of ZnONPs; (**C**) MPsZO-1 microparticles in different aqueous solutions.

**Figure 5:**
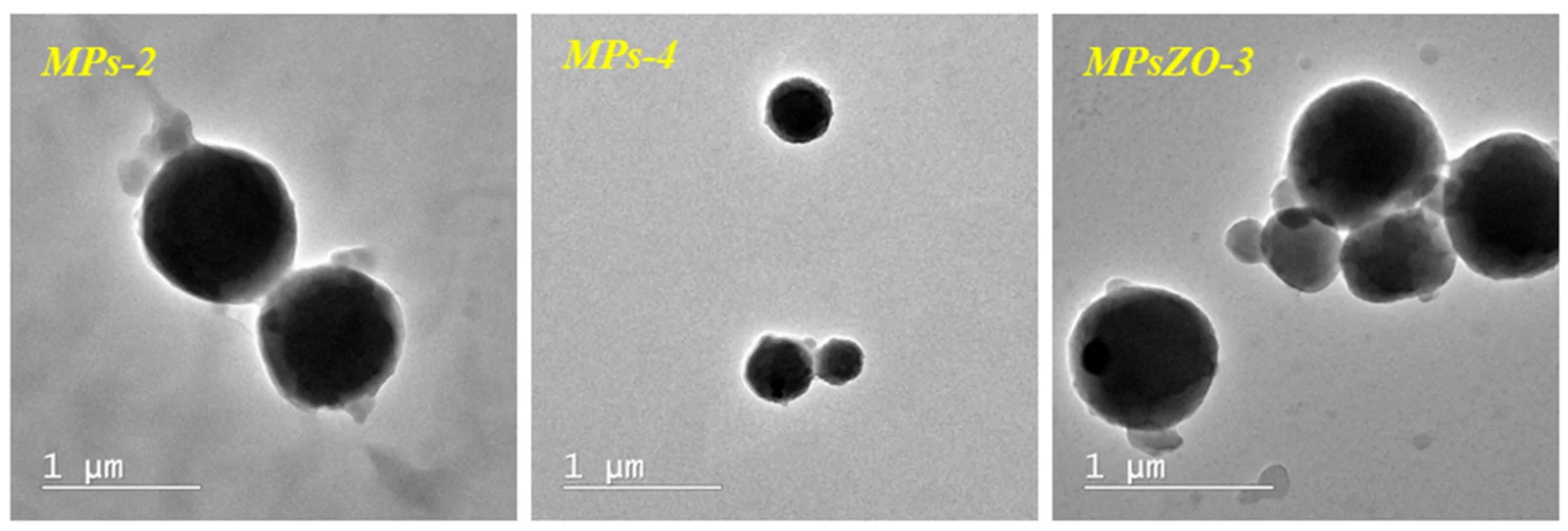
TEM microscopy for microparticle type MPs-2, MPs-4, and MPsZO-3.

**Figure 6:**
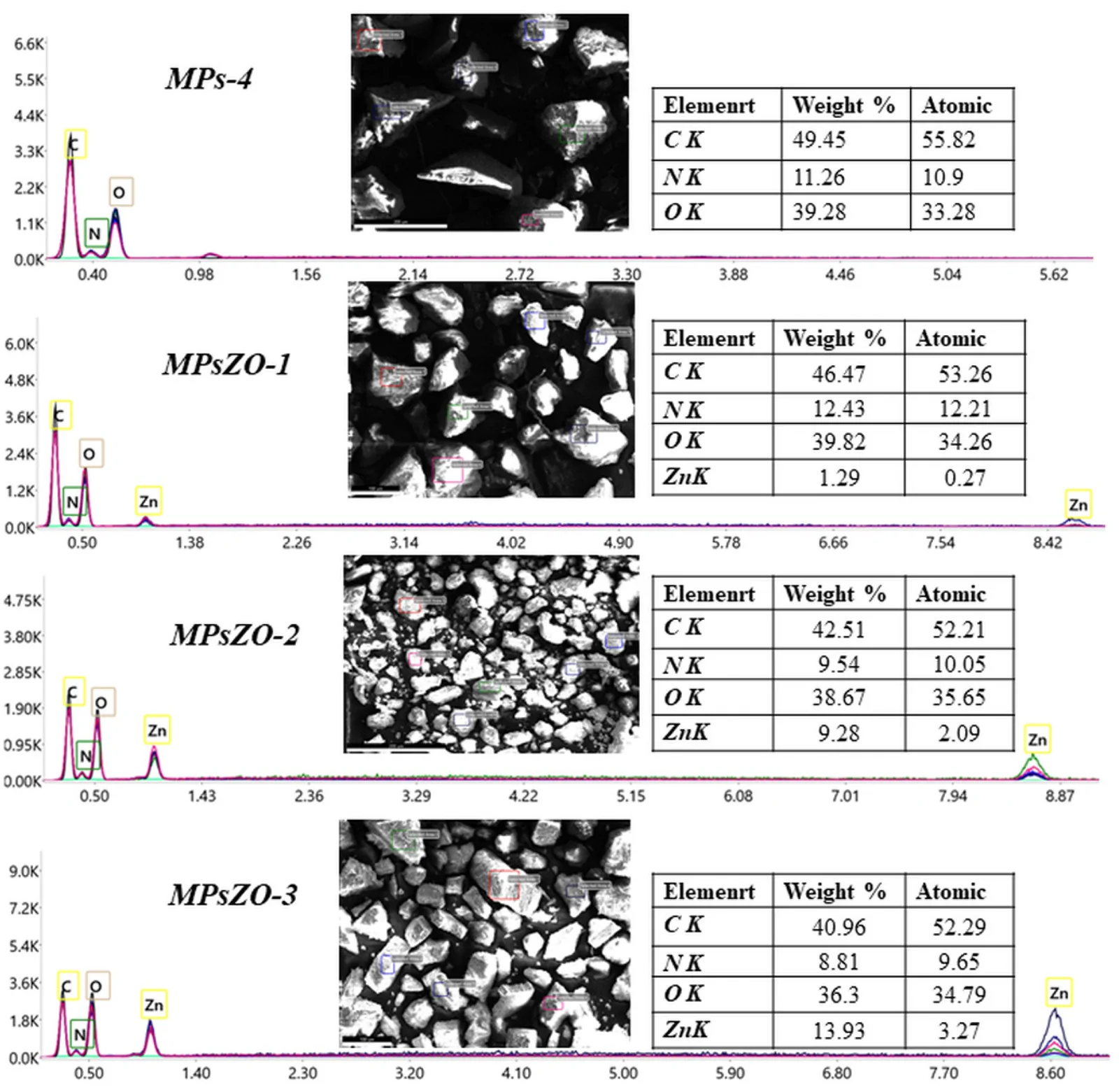
EDAX spectrum for microparticles in the absence and in the presence of ZnONPs.

**Figure 7:**
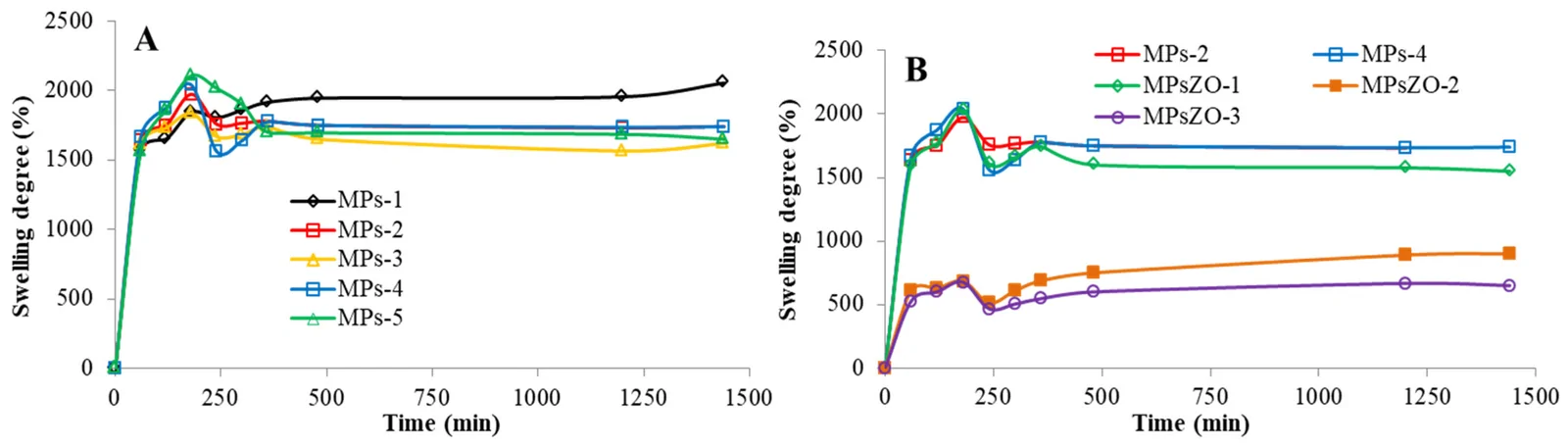
Time-dependent variation of the swelling degree of: (**A**) microparticles without ZnONPs in PBS (pH = 7.4) at 37 °C, (**B**) microparticles in the absence and the presence of ZnONPs in PBS (pH = 7.4) at 37 °C.

**Figure 8:**
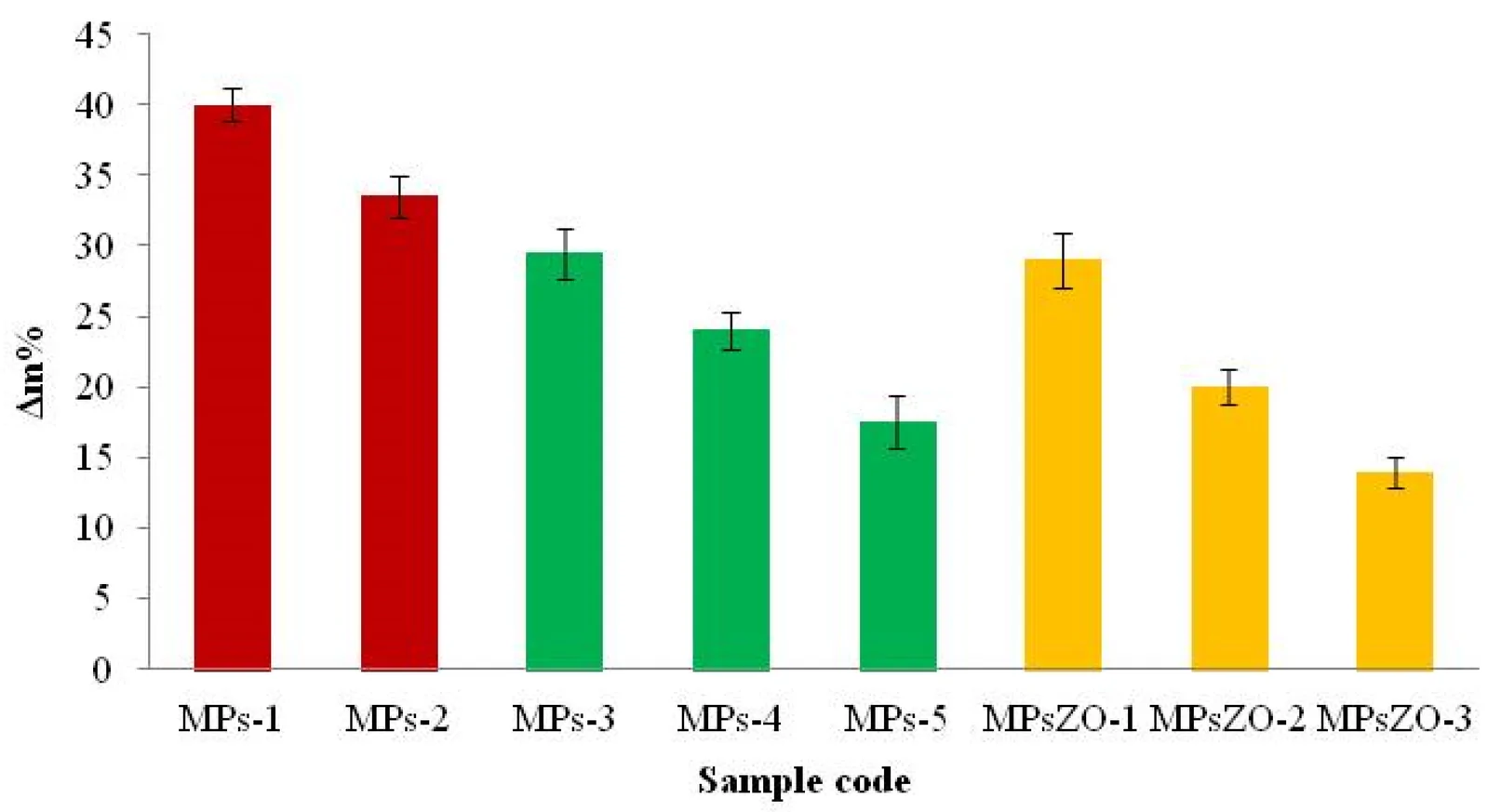
Influence of the ratio between polymers and ZnONPs on the degradation degree of microparticles.

**Figure 9:**
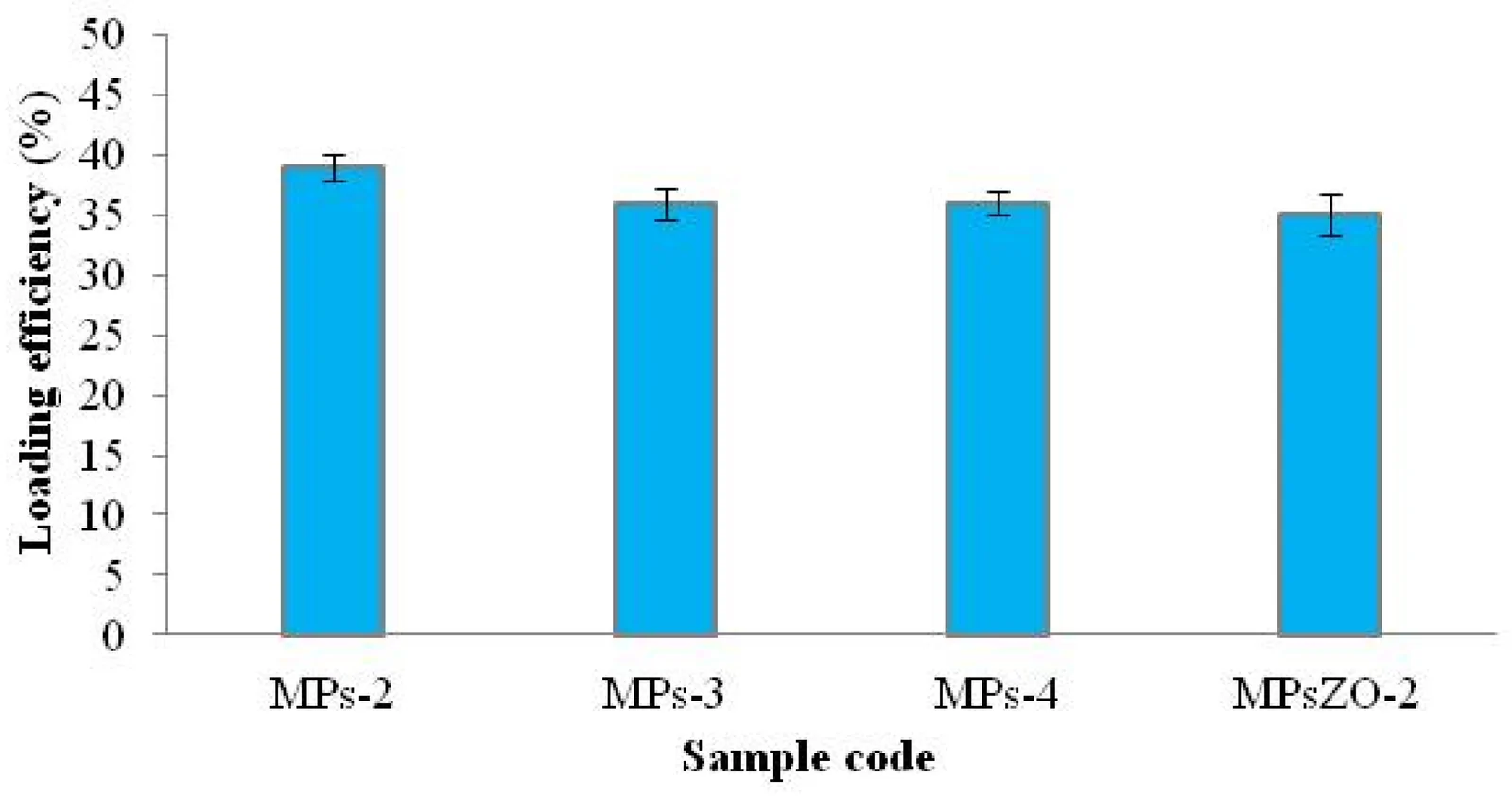
Ibuprofen loading degree in microparticles.

**Figure 10:**
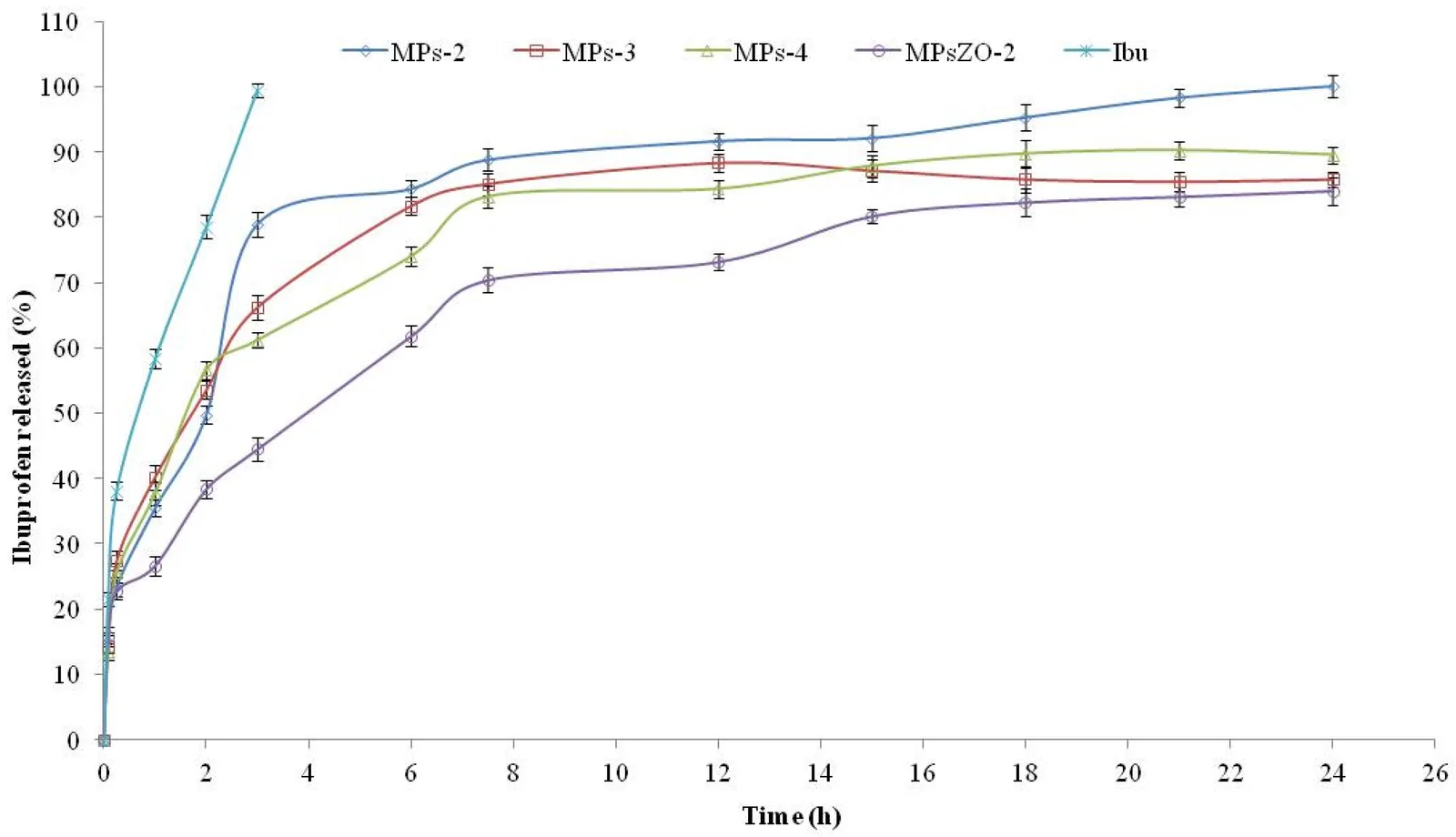
Release profiles of Ibuprofen from microparticles in the absence and in the presence of ZnONPs in PBS (pH = 7.4).

**Figure 11:**
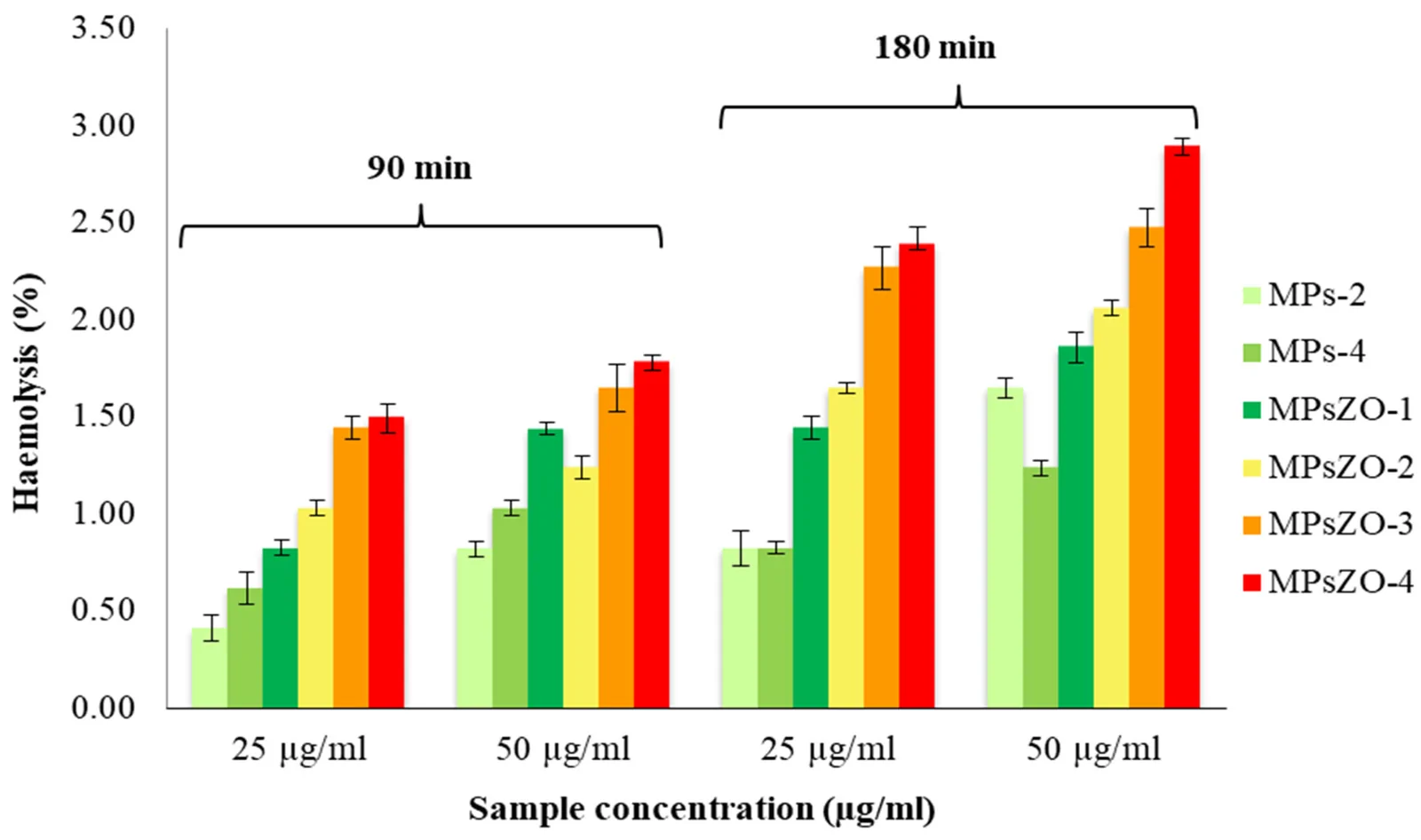
Hemolysis degree after 90 and 180 min of exposure to microparticles in the absence and in the presence of ZnONPs. Data are presented as mean ± SD (n = 3).

**Figure 12:**
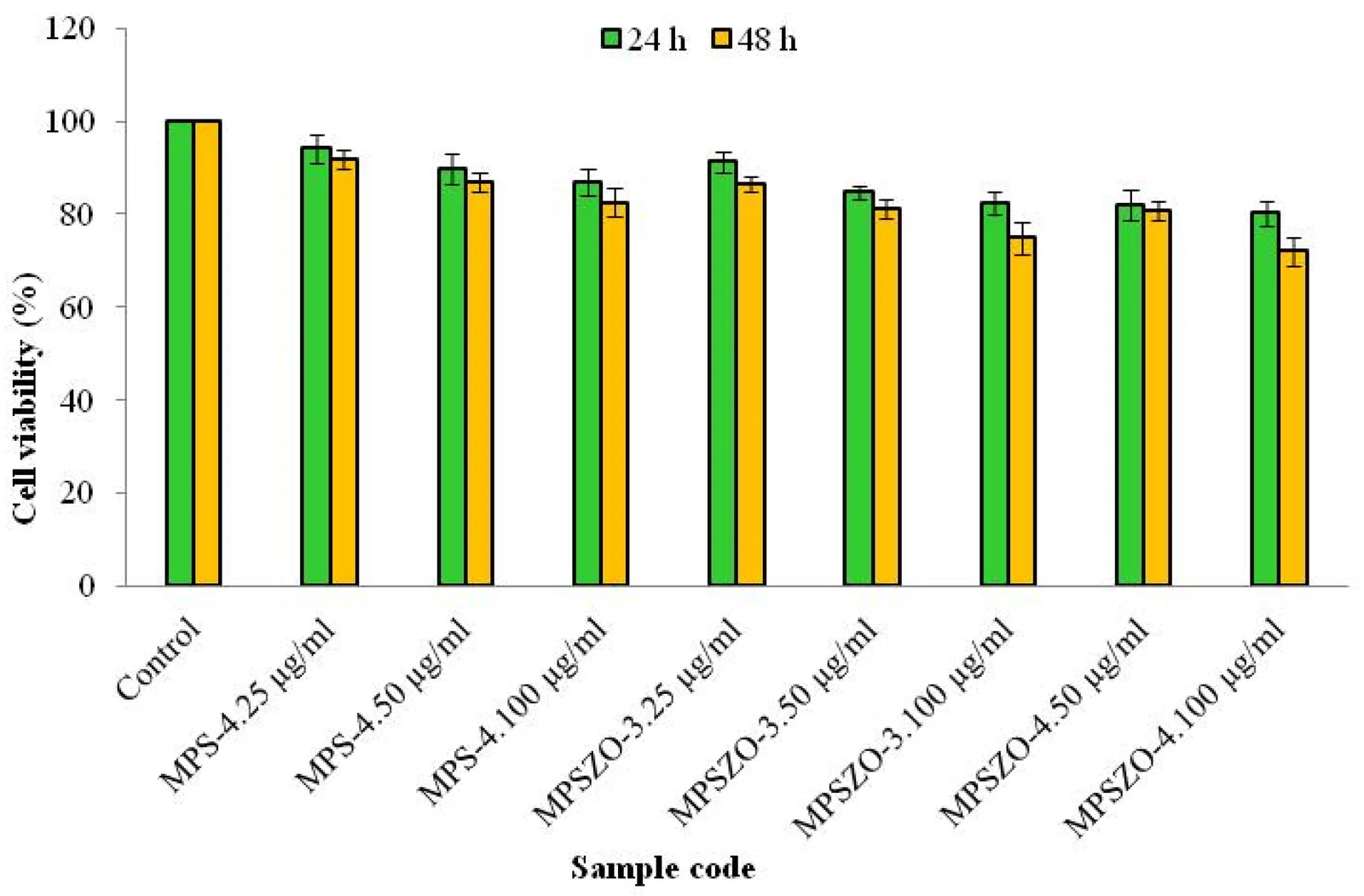
Effect of 24 and 48 h treatment with different concentrations (μg/mL) of microparticles type MPS-4; MPSZO-3 and MPSZO-4 on the viability of human dermal fibroblasts. Data are presented as mean ± SD (n = 3).

**Figure 13:**
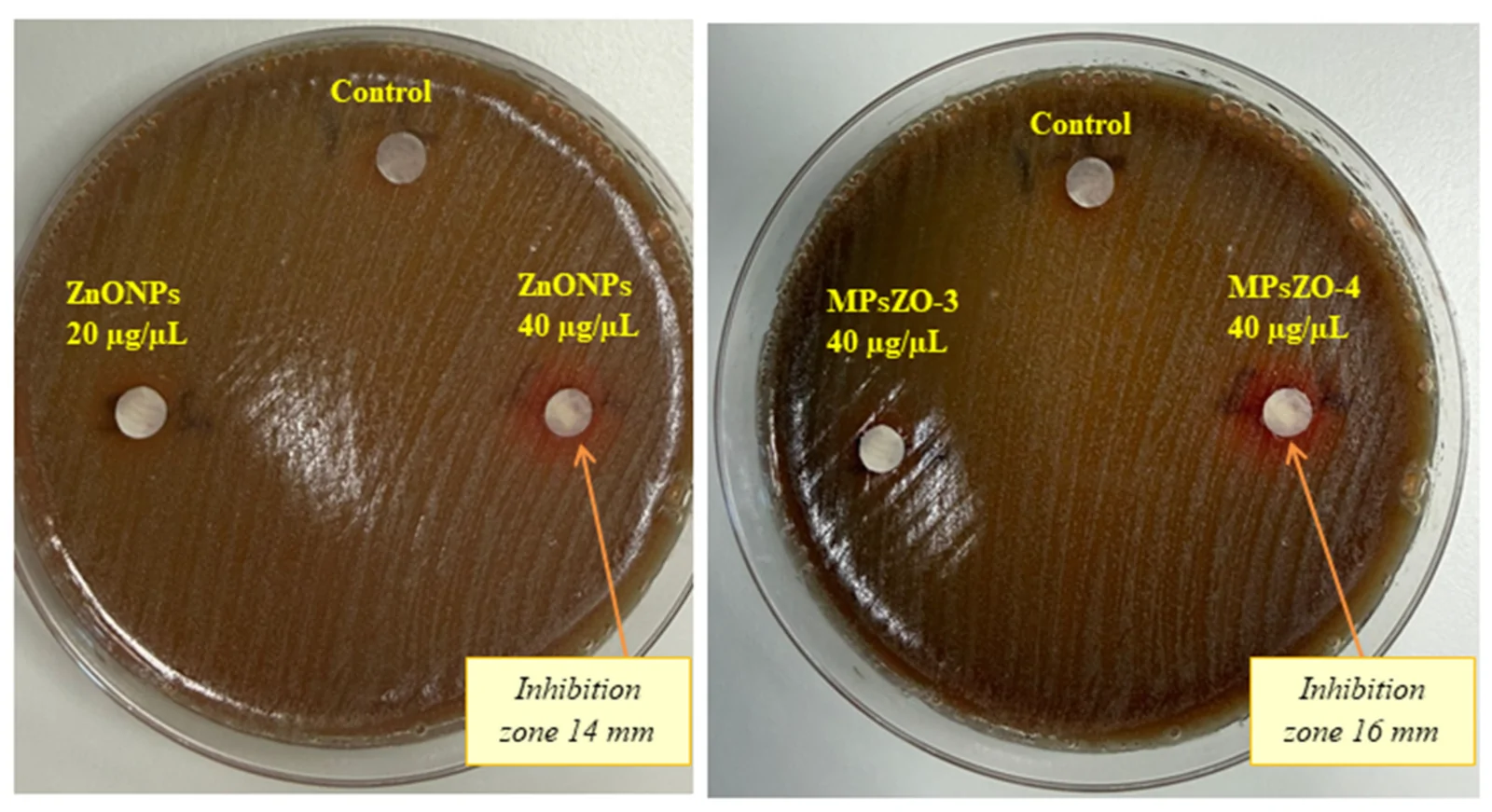
Antimicrobial activity of ZnONPs and of biocomposite microparticles type MPsZO-3 and MPsZO-4.

**Table 1:** Experimental design used for microparticle preparation.

Sample Code	CMCS (mg)	PVA (mg)	Moles of COOH from CMCS/Moles of DMT-MM	ZnO NPs (mg)	DMT-MM (mg)
MPs-1	200	-	2/1	-	539
MPs-2	200	-	3/1	-	808
MPs-3	190	10	3/1	-	768
MPs-4	180	20	3/1	-	727
MPs-5	160	40	3/1	-	646
MPsZO-1	200	-	3/1	6	808
MPsZO-2	200	-	3/1	40	808
MPsZO-3	180	20	3/1	60	727
MPsZO-4	180	20	3/1	130	727

**Table 2:** FTIR characteristic absorption bands of CMCS and microparticles without ZnONPs (MPs-2, MPs-3, and MPs-4) and their corresponding vibrational assignments.

Assignments	CMCS (cm^−1^)	MPs-2 (cm^−1^)	MPs-3 (cm^−1^)	MPs-4 (cm^−1^)	MPS-ZnO-4 (cm^−1^)	ZnO NP (cm^−1^)
O–H stretching	3291.67	3381.75	3371.03	3323.13	3376.03 3288.81	3416.07
C–H stretching	2878.42	2926.33	2887.00	2931.33 2887.00	2926.33	-
Amide I/C=O	-	1646.55	1653.70	1653.70	-	-
O–H stretching	-	-	-	-	-	1501.41
Amide II/N–H; C–N stretching	1582.92	1547.17 1580.77	1578.63 1544.31	1578.63 1545.74	1589.35	-
COO^−^/C–H deformation	1407.04	1467.81	1467.81	1467.81	-	-
O-H bending	-	1372.01	1372.01	1372.01	1399.17	1389.88
C–O/C–O–C stretching	1317.67 1051.70	1312.66 1214.00 1046.70	1312.66 1214.00 1048.84	1313.38 1050.99	1319.81 1056.71	1501.41
C-H bending	711.38	687.08	687.08	685.65	722.11	-
Zn–O vibrations	-	-	-	-	494 426 417	428

**Table 3:** Effect of microparticles composition on mean diameter in microparticles without ZnO (MPs) and with ZnO (MPsZO).

Sample	CMCS/PVA/ZnO (mg/mg/mg)	DMT-MM (mg)	Average Diameter (nm), Mean ± SD	PDI ± SD
MPs-1	200/0/0	539	749.33 ± 63.82 *	0.56 ± 0.08
MPs-2	200/0/0	808	961.67 ± 32.33 *	0.47 ± 0.06
MPs-3	190/10/0	768	664.33 ± 13.87 *	0.45 ± 0.08
MPs-4	180/20/0	727	547.00 ± 17.78 *	0.45 ± 0.04
MPs-5	160/40/0	646	677.33 ± 55.58 *	0.47 ± 0.02
MPsZO-1	200/0/6	808	1121.33 ± 58.86 *	0.59 ± 0.01
MPsZO-2	200/0/40	808	1085.33 ± 39.26 *	0.33 ± 0.06
MPsZO-3	180/20/60	727	791.00 ± 19.67 *	0.57 ± 0.02
MPsZO-4	180/20/130	727	742.33 ± 63.11 *	0.46 ± 0.04

Values are expressed as mean ± standard deviation (SD), n = 3. For the samples without ZnO (MPs-1–MPs-5), differences among samples were evaluated by one-way ANOVA followed by Tukey’s HSD post-hoc test (F(4,10) = 40.4893, *p* < 0.0001(*)). For the samples with ZnO (MPsZO-1–MPsZO-4), one-way ANOVA followed by Tukey’s HSD demonstrated significant differences among samples (F(3,8) = 86.7425, *p* < 0.0001 (*)).

**Table 4:** Zeta potential values of microparticles.

Sample Code	Zeta Potential (mV)		
	Saline Solution	PBS (pH = 7.4)	FBS 10%
MPs-1	−20.0 ± 0.7	−22.5 ± 1.0	−18.5 ± 0.7
MPs-2	−17.6 ± 0.9	−19.6 ± 0.9	−17.3 ± 0.8
MPs-3	−16.1 ± 0.8	−28.2 ± 1.1	−15.9 ± 0.8
MPs-4	−17.2 ± 1.2	−31.0 ± 0.9	−13.8 ± 0.8
MPs-5	−13.3 ± 0.5	−27.7 ± 0.9	−12.1 ± 0.7
MPsZO-1	−17.3 ± 0.4	−22.6 ± 1.0	−8.0 ± 0.8
MPsZO-2	−15.8 ± 0.7	−22.5 ± 0.8	−9.2 ± 0.8
MPsZO-3	−17.5 ± 0.9	−22.0 ± 0.3	−9.9 ± 0.6
MPsZO-4	−15.8 ± 0.5	−21.9 ± 0.7	−14.5 ± 0.4

**Table 5:** Korsmeyer–Peppas parameters for microparticle.

Sample Code	Korsmeyer–Peppas Model			
	Release Rate Constant (k)	Release Exponent (n)	Coefficient of Determination (R^2^)	Release Mechanism
Ibu	0.660594	0.3964	0.9774	quasi-Fickian diffusion
MPs-2	0.37469	0.3657	0.9934	
MPs-3	0.41560	0.3959	0.9739	
MPs-4	0.41071	0.4247	0.9782	
MPsZO-2	0.31025	0.2715	0.9532	

## Data Availability

The original contributions presented in this study are included in the article. Further inquiries can be directed to the corresponding authors.
